# Editorial: *Nucleic Acids Research* annual Web Server Issue in 2016

**DOI:** 10.1093/nar/gkw460

**Published:** 2016-06-17

The 2016 Web Server Issue of Nucleic Acids Research is the 14th in a series of annual issues dedicated to web-based software resources for analysis and visualization of molecular biology data. It is freely available online under NAR's open access policy.

This year, 296 proposals were submitted and 115, or 39%, were approved for manuscript submission. Of those approved, 94, or 82%, were ultimately accepted for publication.

**Topics**. The papers in the Web Server Issue reflect current and emerging trends in bioinformatics and computational biology research. Of particular interest are recent works which move beyond an understanding of bio-molecular phenomena toward an emphasis on the development of engineering tools to exploit that understanding. Examples include CRISPR modification of DNA sequences, functional design of RNA, antibody, and protein structures, and adaptation of metabolic pathways for use as molecular sensors. Seven papers deal with these topics. Also of interest is the maturing of the field of genome annotation which is accompanying the large-scale sequencing and assembly of thousands of non-reference genomes. Six papers describe tools for annotation.

Biomolecular structure remains a strong topic, with 28 papers on protein structure, DNA and RNA structure, and the effects of mutations on structure. Along with these are 13 papers which deal with intermolecular binding, including protein–protein docking, protein–peptide binding, protein–ligand binding, protein-small molecule binding and protein binding to DNA and RNA.

Among the remaining papers, nine describe tools for the identification of differentially expressed protein-coding genes and non-coding RNAs, and tools for enrichment analysis and gene prioritization. Five papers deal with visualization of data, including for phylogenetic analysis and differential expression analysis. Additional topics include metabolic and signaling networks, microRNA analysis, alignment and mitochondrial genome typing.

**Acknowledgements**. The Web Server Issue arises out of the work of many people who I would like to thank. First there are the researchers and scientific programmers who provide us with these outstanding, freely available web resources and who revise and improve their manuscripts and websites under considerable time pressure. Next are the hundreds of referees who conscientiously contribute their time to reviewing and helping improve the manuscripts and websites.

**Figure 1.**
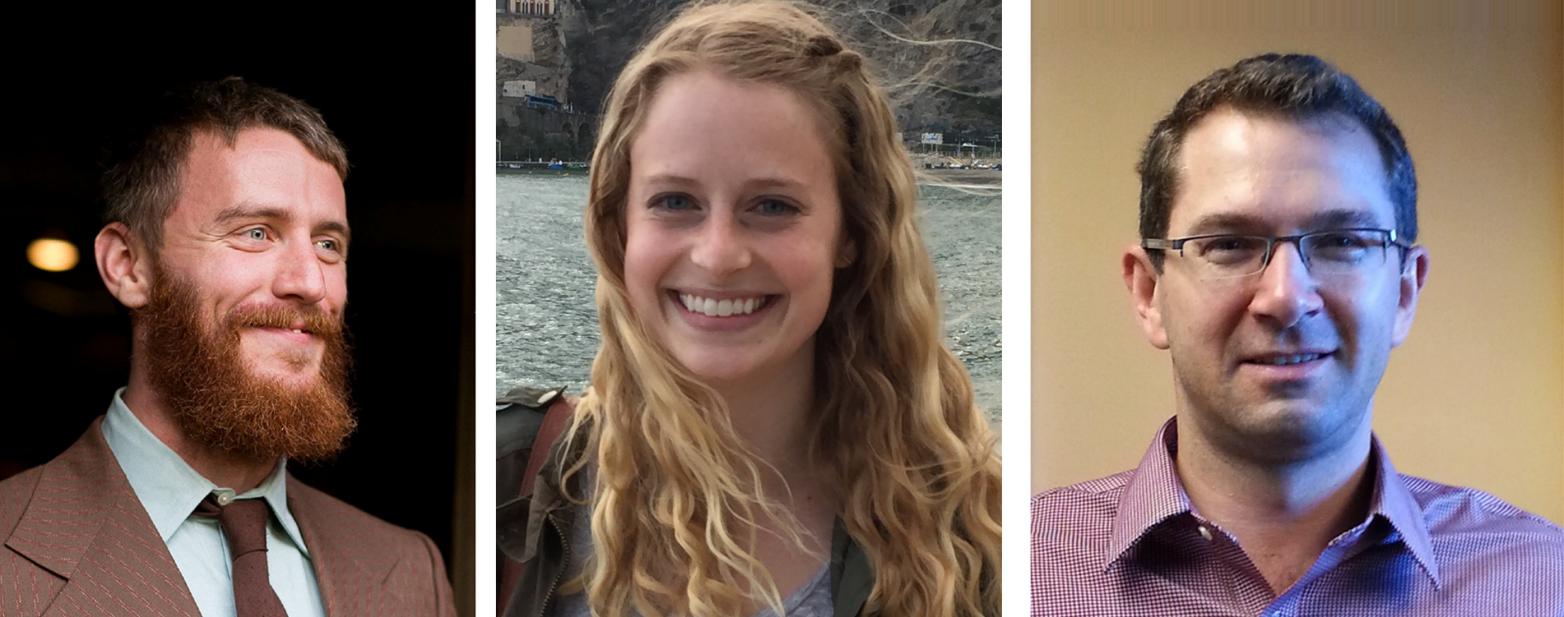
From left: Sean Corbett and Allyson Byrd are PhD students in the Boston University Bioinformatics Graduate Program. Artem Mamonov is a Research Associate in the BU Department of Biomedical Engineering. They provided outstanding assistance in testing the Web Server proposal websites.

My own work on the Web Server Issue is made enormously easier by the dedicated editorial assistance of Fay Oppenheim, who logs the data for all the proposals and interacts with the referees, inviting, cajoling and chasing them so that their reviews get in on time. Thank you. Thanks also to Allyson Byrd and Sean Corbett, PhD students in the Boston University Bioinformatics Program and Artem Mamonov, Research Associate in the Boston University department of Biomedical Engineering, for their hard work in carefully evaluating the proposal websites during the proposal approval phase (Figure 1). Additional thanks to Martine Bernardes-Silva, Editorial Manager, NAR, and Jennifer Boyd and the staff at Oxford University Press.

**Deadlines for 2017**. To streamline the review process, authors are required to send a one-page summary of their web server to the editor, Dr Gary Benson (narwbsrv@bu.edu), for pre-approval prior to manuscript submission. Authors should consult the instructions for summary submission and website design at: http://www.oxfordjournals.org/our_journals/nar/for_authors/submission_webserver.html. For 2017, the summary and URL address of the fully functional website must be submitted by 31 December 2016. The deadline for submission of articles is 31 January 2017.

**Instructions for Submissions**. Review of a proposal includes evaluation of the summary and extensive testing of web server functionality. The key criteria for pre-approval are high scientific quality, wide interest, the ability to do computations on user-submitted data, and a well-designed, well implemented and fully functional website. Note that there is a minimum 2-year interval before publication in the Web Server Issue for web servers, or essentially similar web servers, that have been the subject of a previous publication, including publication in journals other than NAR. With respect to the website, the following are guidelines for approval.

1. It should have an easy-to-find submission page with a simple mechanism for loading test data and setting test parameters. The preferred method is one-click loading. Additional mechanisms that assist the user in submitting data should be implemented where appropriate, for example, automatic download of a pdb structure file once the user has entered the appropriate identifier.
2. Output should be dynamic and rich in detail. Wherever possible, supporting evidence used in calculations and/or links to external databases containing additional information should be provided. Numerical, textual and visual output should be mixed and visualization tools that add information or increase the user's understanding should be utilized. Note that output consisting merely of a few numerical values, a static spreadsheet, or a series of files to be opened in other programs will not be approved. Note also that for security reasons, use of FLASH and Java will no longer be allowed.
3. Web servers that do not finish their calculations immediately must implement a mechanism for returning results to the user. Notification by email may be provided as an option, but an alternative that returns a web link at the time of data submission, which the user can then bookmark and access at a later time, is required. This link should ideally report the status of the job (queued, running, finished). Websites that require a guest login will not be approved. Note that uploaded data and the results of analysis for each user must be private and not viewable by other users.
4. The website should be supported by an extensive help section or tutorial that guides the user through the submission process, contains details about input file formats and parameters, and explains the meaning of the output. Whenever possible, the help pages should link to dynamic output examples similar to those provided by the website. Text and figure help pages, rather than video tutorials, are preferred because they simplify quick look-up.
5. Any proposal for a web server that is *predictive* must include details on validation of predictions from new data not used in training. N-fold cross validation methods will not be considered sufficient. Details should include size and composition of the validation dataset (number of positive and negative cases), and several measures of predictive performance, including sensitivity, specificity and precision. Proposals are frequently rejected for lack of adequate prediction validation information.
6. Websites not clearly designed to accept and analyze user-submitted data will be rejected. This applies to those established primarily for lookup or exploration in a dataset, or serve the function of ‘data aggregators.’ Authors of websites that provide novel data should consider the NAR Database Issue as a possible venue (see the instructions at http://www.oxfordjournals.org/our_journals/nar/for_authors/msprep_database.html).
7. Proposals that describe a new analysis method are generally not appropriate for the Web Server Issue because limited space and the rapid revision process make thorough method description and validation problematic. Authors of such methods might instead consider sending their manuscript to NAR as a regular computational biology paper (see the instructions for authors at http://www.oxfordjournals.org/our_journals/nar/for_authors/criteria_scope.html#Computational%20Biology).

Gary Benson

Executive Editor

Web Server Issue

Nucleic Acids Research

